# Flattening the COVID-19 Curve With Natural Killer Cell Based Immunotherapies

**DOI:** 10.3389/fimmu.2020.01512

**Published:** 2020-06-23

**Authors:** Marisa Market, Leonard Angka, Andre B. Martel, Donald Bastin, Oladunni Olanubi, Gayashan Tennakoon, Dominique M. Boucher, Juliana Ng, Michele Ardolino, Rebecca C. Auer

**Affiliations:** ^1^Cancer Therapeutics Program, Ottawa Hospital Research Institute, Ottawa, ON, Canada; ^2^Department of Biochemistry, Microbiology, and Immunology, University of Ottawa, Ottawa, ON, Canada; ^3^Division of General Surgery, Department of Surgery, University of Ottawa, Ottawa, ON, Canada; ^4^Schulich School of Medicine, University of Western Ontario, London, ON, Canada; ^5^Centre for Infection, Immunity, and Inflammation, University of Ottawa, Ottawa, ON, Canada

**Keywords:** NK cells, COVID-19, innate immunity, immunotherapy, interferon

## Abstract

Natural Killer (NK) cells are innate immune responders critical for viral clearance and immunomodulation. Despite their vital role in viral infection, the contribution of NK cells in fighting SARS-CoV-2 has not yet been directly investigated. Insights into pathophysiology and therapeutic opportunities can therefore be inferred from studies assessing NK cell phenotype and function during SARS, MERS, and COVID-19. These studies suggest a reduction in circulating NK cell numbers and/or an exhausted phenotype following infection and hint toward the dampening of NK cell responses by coronaviruses. Reduced circulating NK cell levels and exhaustion may be directly responsible for the progression and severity of COVID-19. Conversely, in light of data linking inflammation with coronavirus disease severity, it is necessary to examine NK cell potential in mediating immunopathology. A common feature of coronavirus infections is that significant morbidity and mortality is associated with lung injury and acute respiratory distress syndrome resulting from an exaggerated immune response, of which NK cells are an important component. In this review, we summarize the current understanding of how NK cells respond in both early and late coronavirus infections, and the implication for ongoing COVID-19 clinical trials. Using this immunological lens, we outline recommendations for therapeutic strategies against COVID-19 in clearing the virus while preventing the harm of immunopathological responses.

## Introduction

Natural Killer (NK) cells are a key component of the innate immune system and are critical in the response to many viral infections in humans and animal models (1–3). In addition to their beneficial antiviral role, NK cells have also been associated with immunopathology in infections such as respiratory syncytial virus (RSV) (4), influenza A virus (5–8), and hepatitis B (9). Additionally, in the context of non-respiratory viral infections by HIV and HCV, NK cells appear to act as a rheostat by eliminating activated CD4+ and CD8+ T cells, thus preventing T cell-mediated autoimmunity (10). The etiologic agent of the 2019 outbreak of pneumonia in Wuhan, China, was identified as belonging to the *Coronaviridae* family and named Severe Acute Respiratory Syndrome coronavirus 2 (SARS-CoV-2). This virus causes the coronavirus Disease 2019 (COVID-19) which was declared a pandemic by the World Health Organization (WHO) on March 11th, 2020 (11, 12). With the paucity of information currently available, there is a lack of consensus on the role played by NK cells in the response to coronavirus (CoV) infection. In this review, we will explore evidence for both the protective and pathological role that NK cells may play in CoV infection. Based on this knowledge we will comment on immune modulating treatment options that are being developed for the current COVID-19 crisis.

### Coronaviruses and Recent Outbreaks

First discovered in the 1960s, CoVs are part of the *Coronaviridae* family of enveloped positive single-strand RNA viruses (13, 14). The subfamily *Orthocoronaviridae* includes four genera: alphacoronavirus, betacoronavirus, gammacoronavirus, and deltacoronavirus (15). Alpha- and betacoronaviruses circulate in mammals, including bats, gammacoronaviruses infect mostly avian species, and deltacoronaviruses infect birds and mammals (15). Low pathogenic human CoVs (hCoVs), such as HCoV-299E (16), infect upper airways and etiological studies suggest they account for 15–30% of common colds (17, 18). On the other hand, highly pathogenic CoVs infect the lower respiratory tract and can cause severe pneumonia (19). These highly pathogenic CoVs include SARS-CoV-1, the virus responsible for the 2002–2004 Severe Acute Respiratory Syndrome (SARS) epidemic, and MERS-CoV, the virus responsible for the outbreak of Middle Eastern Respiratory Syndrome (MERS) in 2015 (19–21). While highly pathogenic CoVs have become a relatively recent issue for humans; feline, canine, and bovine CoVs have long been recognized as significant pathogens with implications in veterinary medicine and agriculture (22, 23). All CoVs have a roughly 30 kb genome packed into an enveloped helical capsid ranging from 80 to 120 nm (24). At minimum, *Coronaviridae* members encode 4 structural and 16 non-structural proteins (14) with the family owing its name to the crown-like appearance produced by their spike (S) proteins (25). Mutations in the S protein have allowed SARS-CoV1/2 to co-opt ACE2 or MERS-CoV to co-opt dipeptidyl peptidase 4 (DPP4) receptor/CD26 as viral entry receptors, thus facilitating the zoonosis of non-human CoVs (15, 26–28). In addition, another mechanism that may have allowed these viruses to adapt to human hosts is through S protein cleavage by host cell proteases to expose the S2 domain fusion peptide, which induces viral and cellular membrane fusion and results in the release of viral genome into the cytoplasm (15). Genetic sequencing revealed SARS-CoV-2 to be a betacoronavirus that shares 79.0% nucleotide identity with SARS-CoV-1 and 51.8% identity to MERS-CoV (29).

The epidemic of SARS in 2002–2004 caused by SARS-CoV-1 illustrated the devastating potential of coronaviruses to cause serious disease in humans (24). SARS ultimately reached 29 countries and 5 continents causing over 8,000 infections and over 900 deaths. The basic reproductive rate (R_0_) or the number of expected cases arising from one infected individual, ranges from 2 to 4 (20, 30, 31). With its reservoir in bats, SARS-CoV-1 is a zoonosis that was transmitted to humans by palm civets (24, 32, 33). SARS-CoV-1 infects lung pneumocytes (34) and enterocytes in the digestive tract (35) most often producing flu-like symptoms (36, 37). More severe presentations including pneumonia, pronounced lymphopenia, liver abnormalities, and acute respiratory distress syndrome (ARDS) were also reported, with most fatalities due to respiratory failure (19, 36–39).

The subsequent MERS-CoV outbreak in 2015 also originated in bats, with dromedary camels being the intermediary host (14, 40, 41). The R_0_ for MERS-CoV is estimated to be under 1 (21). The extent of MERS-CoV transmission was more limited than SARS-CoV-1, but its case fatality rate was greater with 2,494 cases over 27 countries and 858 deaths being reported at the end of 2019 (21). Common presentations for MERS-CoV include fever, dyspnea, muscle pain, and digestive tract symptoms and disease progression is more likely in those with comorbidities (42).

Like SARS-CoV-1 and MERS-CoV, SARS-CoV-2 is thought to have originated in bats through an unknown intermediary host (43). At the time of writing, the number of global infections is estimated to be over 5,000,000 with over 340,000 deaths (44) and the R_0_ is roughly 2.2 (45). Like other diseases caused by infectious CoVs, most patients present with flu-like symptoms including fever, cough, and lethargy, with the development of pneumonia and ARDS often proving fatal (46). Furthermore, patients with underlying conditions are at risk for further complications if infected with COVID-19, such as those with cardiovascular disease (47). SARS-CoV-2 has been posthumously detected in not only the lungs, but the pharynx, heart, liver, brain, and kidneys (48). Transmission of SARS-CoV-2 is thought to mainly occur through direct contact/inhalation of respiratory droplets and aerosols from infected carriers, but indirect transmission by fomites has also been reported, although less efficient (49, 50). SARS-CoV-2 viral entrance is thought to be mediated by binding of the S protein to the ACE2 receptor (51, 52), although this is still under debate (53). While direct cytopathic effects are thought to play a major role in CoV pathology, studies have suggested that a dysregulated immune response resulting in pathological inflammation is also partly responsible (19). With the current pandemic already surpassing the previous CoV outbreaks (54), rapid deployment of novel approaches to understanding and treating coronavirus infections are needed.

## NK Cells as Innate Viral Killers

### Sensing RNA Viruses

Innate immunity is essential in disease prevention and viral clearance. Among the first responders to viral infections, tissue-resident macrophages and dendritic cells (DCs) (55) recognize evolutionarily conserved microbial structures termed pathogen-associated molecular patterns (PAMPs) via germline-encoded pattern recognition receptors (PRRs) (56). In the context of respiratory RNA viruses, airway epithelial cells, that also express some PRRs (57), are often infected and have a major role in the first line of defense. TLR3, TLR7, TLR8, MDA-5, and RIG-I are PRR expressed by immune and non-immune cells that are especially relevant in fighting respiratory RNA viruses, such as Coronaviruses (57). Sensing through PRRs results in the transcription of genes involved in the inflammatory response, with type I interferons (IFNs) (IFN-α/β) production being a critical part of the antiviral response (58). Type I IFNs are produced by many immune and non-immune cells (55, 57, 59) and in addition to eliciting intrinsic antiviral responses (60), they are also essential to prime innate and adaptive lymphocytes, including NK cells (61).

### NK Cells as Viral Responders

NK cells are cytotoxic lymphocytes that directly target infected, stressed, or transformed cells and play a critical role in bridging the innate and the adaptive immune responses (62). In humans, mature NK cells comprise 10–15% of total peripheral blood leukocytes and are described phenotypically as CD3^−^ CD14^−^ CD19^−^ CD56^+^ CD16^+/−^ (63). NK cells do not undergo clonal selection but instead express several germline-encoded receptors that regulate their activity (62, 64, 65). Upon viral infection, host cells become more susceptible to NK cell killing through: (i) upregulation of self-encoded molecules induced by infection/cellular stress (66, 67) that bind activating NK cell receptors such as Natural Cytotoxicity Receptors (NCRs) (NKp30, NKp44, and NKp46) (68), C-type lectin-like receptors NKG2D and NKp80 (69), and co-activating receptors such as DNAM-1 (70); (ii) downregulation of ligands for inhibitory receptors such as Killer Immunoglobulin-like Receptors (KIRs) (71–73) and the C-type lectin-like receptor CD94-NKG2A (74, 75) which suppress NK cell activation, and; (iii) direct recognition of viral moieties, via engagement of PAMPS (76) or transmembrane activating receptors such as mouse Ly49H (77) or human NKG2C (78). Moreover, NK cells can eliminate virus-infected cells via CD16-mediated antibody-dependent cell-mediated cytotoxicity (ADCC), which has been shown to be particularly important for herpesvirus clearance (79). Finally, NK cell activity is modulated by cytokines, including, but not limited to, the activating cytokines interleukin (IL)-2/12/15/18 (80) and type I IFN, which can be produced by virally infected cells or activated antigen presenting cells (81, 82). IL-2/12/15/18, alone or in combination, promotes NK cell survival, proliferation, cytotoxicity, and cytokine production, including IFN-γ (80). Therefore, NK cells are uniquely equipped to sense and quickly respond to viral infections.

### NK Cell Effector Functions and Memory

NK cells are found in circulation and in peripheral tissues (63) and can be quickly recruited to sites of infection where they facilitate and accelerate viral clearance. In fact, NK cells are not thought to have permanent tissue residency but instead move dynamically between the blood and tissues, such as the lungs (83). NK recruitment is regulated by chemokine gradients that are sensed via chemokine receptors (84, 85). Activated NK cells induce the apoptosis of target cells through the engagement of death receptors, such as TRAIL and Fas (86) or via direct cytotoxicity through Ca^2+^-dependent exocytosis of cytolytic granules (perforin and granzymes) (87). Moreover, NK cells secrete cytokines, including IFN-γ, which have key anti-viral properties (88).

In addition to being essential first responders to viral infection, NK cells can elicit a stronger secondary response resembling the memory features of adaptive lymphocytes (89, 90). NK cell memory has been initially described in mice infected by MCMV, where Ly49H^+^ NK cells quickly expand and have stronger responses after a secondary encounter with the virus (91). Interestingly, a similar NK cell subset has been identified in humans, where NK cells expressing NKG2C are expanded and persist in CMV infected patients (92). Both Ly49H and NKG2C bind viral determinants, highlighting how NK cell memory is linked with the ability of NK cells to directly recognize viruses (93, 94). In addition to direct recognition of viral molecules, long-lasting changes in NK cells are induced by the cytokine milieu (89, 95), which can be elicited by viral infection.

### NK Cell Dysfunction Is Linked With Increased Viral Susceptibility

The relevance of NK cells in fighting viral infections has been highlighted by several studies where NK cells, in mice and humans, were not present or had compromised functions (96). For example, individuals with NK cell deficiencies (NKD), a subset of primary immunodeficiency diseases, are highly susceptible to viral infection, particularly by herpesvirus and papillomavirus families (96). The seminal 1989 case of NKD in an adolescent female with severe herpesvirus infections (varicella pneumonia, disseminated CMV, and disseminated HSV) revealed how functional NK cell deficiencies have clinical consequences in terms of viral infections (97). Cancer patients are also at risk of viral infections (98), which may be explained, at least in part, by an impairment of NK cell responses often observed in humans and in murine tumor models (99–104).

Unsurprisingly, cancer patients are at a significantly increased risk of severe COVID-19 (105, 106). Elderly patients are also more susceptible to viral infections (107). Mouse studies highlighted how a decreased number of circulating mature NK cells in aged animals paralleled with increased susceptibility to viral infections (108). Studies in humans suggest that although NK cell numbers can actually increase with aging, NK cell activity declines significantly (109, 110). Przemska-Kosicka et al. investigated NK cell function in response to seasonal influenza vaccination in young and old populations and observed quantitative and qualitative changes associated with impaired responses in the NK cell population and this was associated with poor seroconversion in the older population (111). Additionally, obesity, which has been shown to cause systemic NK cell dysfunction (112, 113), has also been linked to increased COVID-19 severity and could be the reason behind the high prevalence of severe COVID-19 in younger people (113). In short, NKD and individuals with reduced NK cell numbers or function are more susceptible to viral infections. Unsurprisingly, the CDC has already highlighted a higher risk of infection and severity of COVID-19 in older individuals and individuals with comorbidities such as obesity and cancer (114). However, this point is still controversial as a systematic review showed that primary immunodeficiencies are not linked with increased COVID-19 severity (115), but these data have to be interpreted keeping in mind that a large part of COVID-19 pathology is caused by excessive immune activation, which is arguably harder to reach in immunocompromised individuals. Given the paradoxical role of the immune response in COVID-19 patients, it would be extremely useful to be able to rely on immunological functional biomarkers that could predict the outcome of disease severity. Such assays are readily available for determining NK cell activity, e.g., NKVue™, and there is therefore an opportunity to conduct studies that would link NK cell functions to disease severity.

## NK Cells and Coronavirus Infections: Dual Roles

### Coronaviruses Potently Suppress Type I IFN Responses

Evasion of host immune responses is necessary for the successful propagation of a virus. Mechanisms employed by CoVs to evade the immune response could provide insights into how the immune system, and NK cells in particular, responds to SARS-CoV-2. CoVs have been shown to target components of the innate IFN response, employing non-structural proteins (nsps), structural proteins, and accessory proteins to achieve this goal. Nsp16 methylates viral RNA therefore preventing recognition by MDA5 and dampening type I IFN production (116). Nsps also suppress type I IFN responses via the inhibition of the transcription factor STAT1 mRNA transcription (nsp1) and deubiquitination of transcription factors like Interferon Regulatory Transcription Factor (IRF)3 (nsp3) (116). Moreover, viral-encoded accessory proteins from SARS-CoV-1 open reading frame (ORF)3b and MERS-CoV ORF4a/4b also block IFN production and signaling (116). In addition, the MERS-CoV ORF6-encoded protein blocks p-STAT1 import, thus blocking IFN signaling (116). Finally, the structural M protein of MERS-CoV (27) physically sequesters kinase proteins RIG-I, TBK1, IKKe, and TRAF3 and the SARS-CoV-1 N protein inhibits Activator Protein (AP)-1 signaling, protein kinase R function, and NFκB activation, all of which act to impede IFN responses (117). *In vivo* murine studies report young mice rapidly clear SARS-CoV-1 infection, while old mice do not and that this discrepancy is due to a delay in type I IFN. Furthermore, early administration of IFN-β induces a stronger immune response and reduces mortality in old mice (118). Since type I IFNs are critical for NK cell activation and effector functions, it is possible that NK cell-mediated clearance of SARS-CoV-2 is being subverted by these mechanisms. Further research into the role of NK cells in CoV clearance and potential immune evasion mechanisms are necessary to inform therapeutic development and use.

### NK Cell Role in Clearing Acute Coronavirus Infections

There is currently a paucity of studies into the role of NK cells not only in COVID-19 pathophysiology, but also in other coronavirus infections. An *in vivo* study reported that beige mice on a B6 background cleared SARS-CoV-1 normally, indicating that functional lymphocytes, including NK cells, may not be required to eliminate SARS-CoV-1 in murine models (119). However, in a more recent study characterizing the cellular immune response to SARS-CoV-1 in 12–14-month old BALB/c mice, T cell depletion did not prevent control of SARS-CoV-1 replication (120), suggesting a role for the innate immune system, and NK cells, in viral clearance. Importantly, in this study CD4-depletion resulted in enhanced lung immunopathology and delayed viral clearance, while CD8-depletion did not affect viral replication or clearance, thus highlighting an important role for CD4^+^ T cells in coronavirus infection. These conflicting results may be due to the inherent limitations of CoV murine models. In 4–8 week-old mice, SARS-CoV-1 is associated only with mild pneumonitis and cytokines are not detectable in the lungs (119, 121, 122). A SARS-CoV-1 isolate (MA-15) replicates to a high titer and is associated with viremia and mortality, however the model lacks significant inflammatory cell infiltration into the lungs (123). Thus, mouse models developed for the study of SARS fell short in terms of reproducing the clinical and histopathological signs of disease (119, 121–123). It is therefore necessary to develop a usable animal model that is capable of reproducing the clinical and histopathological signs on COVID-19. Israelow et al. recently described a SARS-CoV-2 murine model based on adeno associated virus (AAV)9-mediated expression of human (h)ACE2, which replicated the pathologic findings found in COVID-19 patients (124). This model, which overcame the inability of murine (m)ACE2 to support SARS-CoV-2 infection, was used to show the inability of Type I IFN to control SARS-CoV-2 replication (124). In a similar attempt to overcome the lack of infectability through mACE2, Dinnon et al. recently described a recombinant virus (SARS-CoV-2 MA) with a remodeled S protein mACE2 interface, which replicated in upper and lower airways in young and aged mice with disease being more severe in aged mice. The authors used this model to screen therapeutics from vaccine challenge studies and assessed pegylated IFN-λ-1 as a promising therapeutic. The authors suggested that this model has greater ease of use, cost, and utility over transgenic hACE2 models (125) to evaluate vaccine and therapeutic efficacy in mice (126).

A preliminary analysis of NK cell function and phenotype has been performed by Zheng et al. using peripheral blood from COVID-19 patients (127). On admission, NK cell levels in the peripheral blood inversely correlated with disease severity. Furthermore, COVID-19 patients with severe disease had significantly lower numbers of circulating NK cells, as compared to mild disease (*p* < 0.05) (127). Additionally, circulating NK cells in severe disease displayed increased expression of the inhibitory receptor NKG2A and had an hyporesponsive phenotype with lower levels of IFN-γ, tumor necrosis factor (TNF)-α, IL-2, and granzyme B, although degranulation was maintained (127). Finally, as compared to patients with active disease, patients recovering from COVID-19 had higher numbers of NK cells and lower NKG2A expression (127). Liao et al. performed single-cell RNAseq on the cells obtained from bronchoalveolar lavage fluid of severe and mild COVID-19 patients and found that COVID-19 patients had significantly more NK cell infiltrates into the lungs, however patients with severe disease had reduced proportions of NK cells (128). In addition, *KLRC1* (NKG2A) and *KLRD1* (CD94) were highly expressed by NK cells (128). Carvelli et al. analyzed myeloid and lymphoid populations by immunophenotyping from blood and bronchoalveolar lavage fluid (BALF) in 10 healthy controls, 10 paucisymptomatic COVID-19 patients, 34 pneumonia patients, and 28 patients with ARDS due to SARS-CoV-2 and found that absolute numbers of peripheral blood lymphocytes, including NK cells, were significantly reduced in the pneumonia and ARDS groups compared to healthy controls. Furthermore, the proportion of mature NK cells was reduced in patients with ARDS and NK cells showed increased NKG2A, PD-1, and CD39 (129). Finally, Wilk et al. performed single-cell RNA-sequencing on 7 COVID-19 patients and 6 healthy controls and found that the CD56^bright^ population was depleted in all COVID-19 patients but the CD56^dim^ population was depleted only in patients with severe COVID-19. Furthermore, NK cells had increased expression of the exhaustion markers LAG3 and HAVCR2 (130). NK cell cytopenia seems to be a consistent characteristic among SARS-CoV-2 infected patients (131). Altogether, these data indicate alterations in the NK cell phenotype and functional profile that are consistent with the hypothesis that to establish a productive and lasting infection, SARS-CoV-2 needs to dampen the NK cell response.

NK cell dysfunctions were also observed in patients from the previous CoV outbreaks. Wang et al. assessed NK cell number and phenotype using peripheral blood from 221 SARS patients admitted to hospitals in Beijing, China (132). NK cell proportion and absolute number were significantly reduced in SARS patients as compared to healthy donors and patients infected with the bacterium *Mycoplasma pneumoniae* (131). NK cell number correlated inversely with disease severity and patients with anti-SARS CoV-specific IgG or IgM antibodies had significantly fewer NK cells (132). The patients assessed had varied disease duration from 4 to 72 days (mean 31.7 days) and this allowed for patient stratification by disease duration. Within the first 10 days of SARS-CoV-1 infection, NK cell numbers remained high but this period was followed by the development of lymphopenia with levels recovering only around day 40 (132). Dong et al. also observed a reduction of NK cell numbers in SARS patients, and these levels were lower in patients with severe, as compared to mild, SARS (133). In addition, MERS infection is strongly associated with leuko- and lymphopenia (42, 134–136).

The mechanisms underlying the reduction of circulating NK cells in patients infected with CoVs are still unclear. As most studies have focused on peripheral blood NK cells, it is possible that the reduced number of circulating NK cells is due to redistribution of blood NK cells into the infected tissues (137). While it is hard to assess NK cell migration to infected tissues in COVID-19 patients, this hypothesis was corroborated by mouse studies, where NK cells have been shown to migrate to the lungs in CoV infected animals (120).

An abundance of inhibitory factors, such as TGF-β, may be partially responsible for the NK cell hyporesponsiveness observed in COVID-19 patients. In support of this hypothesis, Huang et al. found significantly higher TGF-β levels in SARS patients compared to healthy controls and this positively correlated with length of stay (138). Given the importance of TGF-β in suppressing NK cell functions, it is possible that the higher levels of TGF-β (as well as other inhibitory cytokines) in CoV patients leads to suppression of NK cell antiviral activity (138). Early studies of COVID-19 patients report secondary (super-) infections, including nosocomial pneumonia or bacteremia as a complication of SARS-CoV-2 infection (138). Since NK cells are critical first responders that play a role in preventing and clearing infections (139), a poor NK cell count or exhausted phenotype, in addition to negatively influencing COVID-19 patient outcomes, could facilitate the development of secondary infections and have a significant negative impact on patient outcomes.

One of the main barriers in studying the role of NK cell activation in the early clearance of CoV infection in asymptomatic or mildly symptomatic patients is the fact that these individuals are rarely diagnosed in the clinic and therefore an opportunity to collect samples for research does not exist. Thus, while there is currently no direct evidence to support a role for NK cells in the clearance of SARS-CoV-2, evidence showing that viral infection has a negative effect on the NK cell compartment is accumulating. Given the importance of NK cell activity in early viral clearance and late immunopathology, having a rapid and reliable test to predict NK cell function, such as NKVue™ (ATGen Canada/NKMax), whereby whole blood is stimulated by an NK cell-specific activating cytokine mix and activity is measured via IFN-γ production, might allow researchers to predict who will mount an adequate response with asymptomatic or minimally symptomatic viral clearance and who will need ICU admission, as has been shown with cancer patients (140). Further research will be required into the innate immune response to CoV infection to more fully understand NK cell contributions to viral clearance.

### NK Cell Role in Coronavirus Immunopathology

In the context of CoVs, the significant morbidity and mortality associated with severe disease is due to acute lung injury (ALI) and the development of ARDS (19, 141). Pathological analysis of tissues obtained from SARS and MERS patients showed edematous lungs with areas of consolidation, bronchial epithelial denudation, loss of cilia, squamous metaplasia, pneumocyte hyperplasia, and bronchial submucosal gland necrosis (19, 29). Histological features include diffuse alveolar damage and acute fibrinous and organizing pneumonia (29). A heightened inflammatory response in the lungs resulting in tissue damage has been hypothesized to explain the development of ALI.

There are several key factors that may be responsible for the induction of this dangerous inflammation (138). Both SARS-CoV-1 and MERS-CoV replicate to high titers early in infection, which could lead to enhanced cytopathic effects and increased production of pro-inflammatory cytokines/chemokines by infected cells. Chen et al. developed a pneumonia model where pulmonary replication of SARS-CoV-1 was associated with histopathological evidence of disease, including bronchiolitis, interstitial pneumonitis, diffuse alveolar damage, and fibrotic scarring (120). They identified a biphasic cellular immune response in which cytokines (TNF-α and IL-6) and chemokines [interferon gamma-induced protein (IP)-10, monocyte chemoattractant protein (MCP)-1, macrophage inflammatory protein (MIP)-1a, RANTES] were produced early, likely by infected airway epithelial cells, alveolar macrophages, and recruited inflammatory monocyte-macrophages and neutrophils, which have been shown to replace resident alveolar macrophages (19, 142). SARS-CoV-1 and MERS-CoV encode structural and non-structural proteins that antagonize the interferon response, which may initially delay the innate immune response but eventually potentiate inflammatory monocyte-macrophage responses (19). In COVID-19 patients, Liao et al. reported increased lung infiltration by macrophages identified via RNA-seq analysis of bronchoalveolar lavage fluid. Patients with mild cases exhibited infiltration by alveolar macrophages [Fatty Acid Binding Protein (FABP)4^+^] while patients with severe ARDS exhibited infiltration by highly inflammatory [Ficolin (FCN1)^+^] monocyte-derived macrophages (128).

In the SARS-CoV-1 pneumonia model, the first wave of cytokines and chemokines induced an accumulation of NK cells, as well as plasmacytoid (p)DCs, macrophages, CD4^+^ T cells and NKT cells in the lungs. A second wave of inflammatory mediators was detected later on day 7 post-infection [cytokines TNF-α, IL-6, IFN-γ, IL-2, IL-5, and chemokines MCP-1, MIP-1a, RANTES, monokine induced by gamma interferon (MIG), IP-10] and correlated with lung infiltration of T cells and neutrophils (120). These findings are consistent with studies that have shown increased levels of activating and inhibitory cytokines and chemokines in the blood and lungs of SARS patients, as well as histological studies of SARS and MERS-infected lungs which show extensive cell infiltrates (19, 29, 143–145). When Huang et al. investigated the cytokine/chemokine profile in the acute phase of SARS infection in a cohort of Taiwanese patients, they observed an IFN-γ-led cytokine storm (138). They assessed sera from hospitalized patients prior to the administration of immunomodulators and found significantly increased levels of IFN-γ, IL-18, IP-10, MCP-1, MIG, and IL-8 (138), which returned to basal levels in convalescent sera. IP-10, MIG, MCP-1, and IL-18 levels were all significantly increased in death vs. survival groups. Interestingly, they found an inverse relationship between IFN-γ levels and lymphocyte numbers and suggested this could either be due to IFN-γ-induced lymphocyte apoptosis or sequestration of chemokine-recruited lymphocytes in the lungs (138). Indeed, this hyper-cytokinemia has been consistently observed in SARS-infected patients (146). However, a recent study found that levels of six pro-inflammatory cytokines (IL-1b, IL-1Ra, IL-6, IL-8, IL-18, and TNF-α) implicated in the cytokine storm in COVID-19 patients did not differ significantly from levels in cytokine storms caused by other conditions. They suggest that it is therefore possible that increased levels of pro-inflammatory cytokines in the context of severe COVID-19 may simply reflect an increased viral burden rather than an exuberant immune response and suggest that immunotherapies should therefore be used with caution (147).

**Figure 1:**
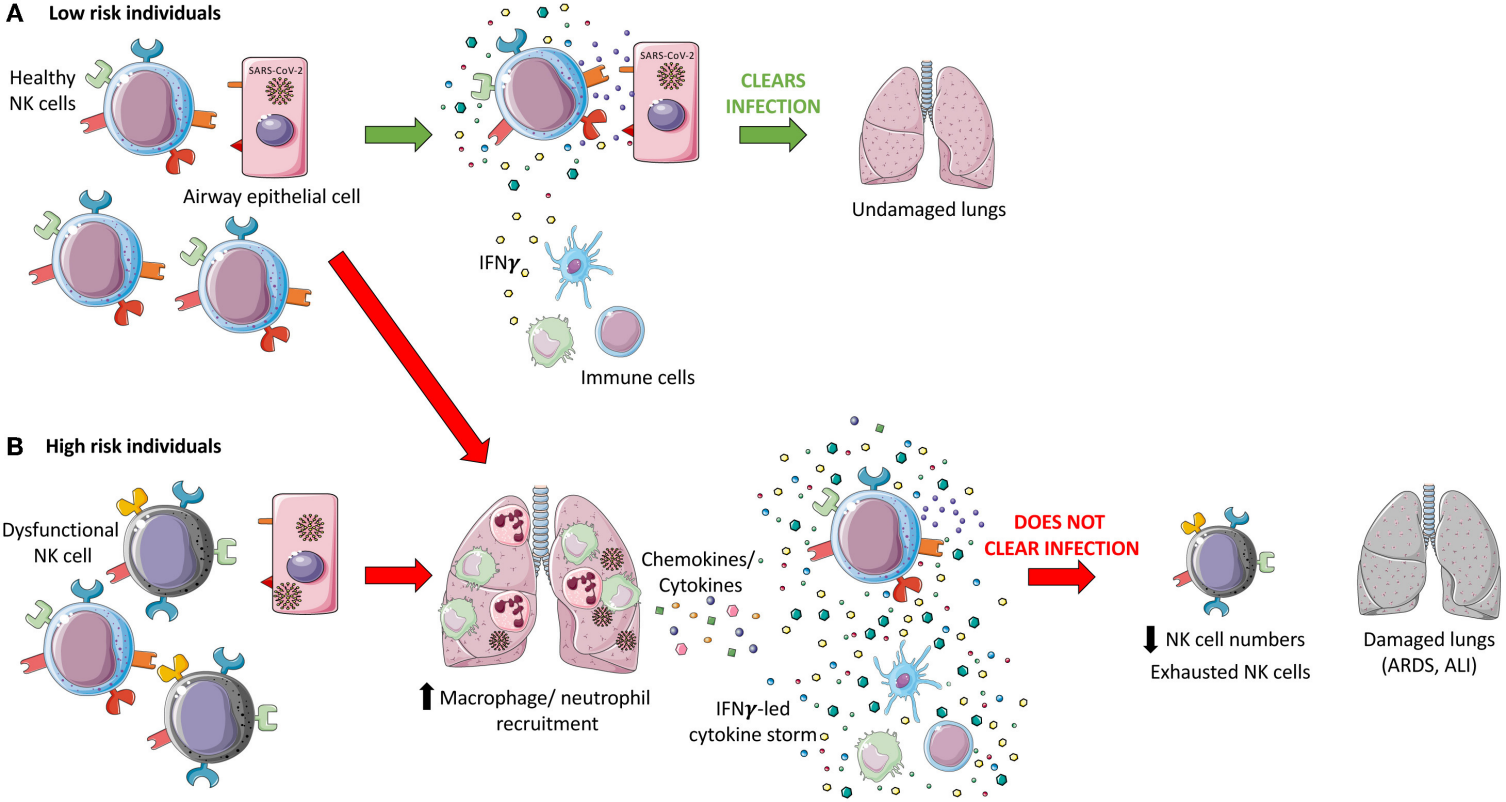
Hypothesized dual role of NK cells during coronavirus pathogenesis. **(A)** Healthy Natural Killer (NK) cells in low-risk individuals recognize SARS-CoV-2 infected cells via recognition of viral proteins on the surface of infected cells and through sensing of cytokines and chemokines produced in response to infection. These cells are hypothesized to be able to directly induce apoptosis through death receptor ligation, antibody-dependent cell-mediated cytotoxicity (ADCC), and through the release of cytotoxic granules, in addition to indirectly targeting virally infected cells via modulation of the immune response through cytokine secretion. An effective innate immune response may be able to clear SARS-CoV-2 infection and leave the patient's lungs undamaged. **(B)** High risk individuals may have dysfunctional NK cells which may not recognize and respond to SARS-CoV-2 infection due to immune evasion strategies employed by the virus. It is hypothesized that an accumulation of infected epithelial cells and innate immune cells, monocyte-macrophages and neutrophils, release cytokines, and chemokines which further recruit immune cells, including NK cells, to the lungs. This may result in the induction of a cytokine storm, led by IFN-γ. This inflammatory state could act as the catalyst for the development of acute lung injury (ALI) and acute respiratory distress syndrome (ARDS), contributing to the significant morbidity, and mortality associated with COVID-19. SARS-CoV-2 infection is associated with reduced NK cell levels and an exhausted phenotype which may impede viral clearance, in addition to severe lung damage.

Altogether these studies show that during acute CoV infection, inflammatory monocyte-macrophages and neutrophils accumulate in the lungs and produce cytokines and chemokines that induce the activation and migration of lymphocytes, including NK cells, to the lungs, where they could be one of the main producers of IFN-γ (148). Under normal conditions, human lung NK cells are typically hyporesponsive but dynamically migrate in and out of pulmonary tissues (83). This supports the hypothesis that during infectious respiratory diseases, an increased recruitment of hyperresponsive NK cells would worsen the festering immunopathology (8). In fact, through Viral-Track scanning of unmapped single-cell RNA-sequencing data, Bost et al. showed that patients with severe COVID-19 exhibited a hyperinflammatory response with an enriched and highly proliferative NK cell compartment (142). High levels of IFN-γ leads to epithelial and endothelial cell apoptosis and vascular leakage, suboptimal T cell response, accumulation of alternatively activated macrophages and altered tissue homeostasis, and ARDS (19), all of which may contribute to COVID-19 disease severity. In summary, the evidence is consistent with the hypothesis that NK cells are involved in the cytokine storm associated with CoV infection and that this hyper-cytokinemia contributes significantly to disease severity via inflammation-mediated lung damage (Figure 1).

Interestingly, this duality of NK cell roles mirrors what is seen in critically ill patients with sepsis. Studies suggest that while early NK cell stimulation and IFN-γ production is beneficial to combat infections, excessive and prolonged stimulation of NK cells leads to reduced NK cell numbers and an exhausted phenotype and was associated with increased systemic inflammation in systemic inflammatory response syndrome (SIRS)/sepsis and increased mortality (149–152).

This review of the literature suggests that NK cells may play an important role in both CoV clearance and immunopathology. The continued probing of NK cell involvement is essential for a more complete understanding of CoV pathophysiology and for the deployment of immunotherapeutics. Depending on the patient, the stage of disease, and other still poorly understood factors, it may be necessary to either boost NK cell activity to ensure viral clearance, e.g., at exposure or during early infection, or to finely tune NK cell effector functions in late stage infections to prevent hyper-cytokinemia and inflammatory lung damage. Indeed, all CoVs that infect humans are zoonoses and there is an extensive reservoir of CoVs that could serve as a source for future pandemics (14, 153). Therefore, a broader understanding of the immune response to coronaviruses and insights into therapeutic implications will be of significant value not only for the current COVID-19 pandemic, but also for potential future pandemics.

## Flattening the Curve With Natural Killer Cells

The race to vaccinate and find a cure for COVID-19 has resulted in a spectacular effort from researchers and medical practitioners around the world. Early attempts at creating targeted therapeutics have mostly relied on historical evidence from related, but not identical, coronaviruses and on the paucity of studies investigating SARS-CoV-2. These strategies have attempted to combat the virus by targeting various stages of its life cycle starting with neutralizing SARS-CoV-2 virions using monoclonal antibodies or plasma from convalescent patients (154). The entry mechanism of CoVs has been shown to rely on binding the ACE2 receptor and using proteases such as TMPRSS2 for S protein priming (52). Thus, preventing ACE2 receptor binding through blocking antibodies or competitive binding with soluble ACE2 and TMPRSS2 protease inhibitors (Camostat mesylate) are being tested (155). Upon viral entry, the viral proteolysis or replication cycle can be targeted with protease inhibitors (Lopinovir and Ritonavir) (156) or RNA-dependent RNA polymerase inhibitors (Remdesivir and Ribavirin) (157). At the time of writing this review, the results of these trials have not been released or are still preliminary and will require further evaluation to assess their clinical efficacy in larger cohort studies. As NK cell activity is critical for viral clearance and may be involved in disease immunopathology, a rapid and reliable predictor of NK cell function may allow for the prediction of clinical progression and the stratification of patients to receive therapeutic intervention. The remainder of this review will discuss the various ways immunotherapies are being deployed to tackle COVID-19, with a focus on therapies that use NK cells (Table 1). Lastly, while NK cells play an important role in combating viral infections, we also need to be fully cognizant of the potential damage immunotherapies could have in severe cases of COVID-19, and how these adverse effects may need to be attenuated (Table 2).

**Table 1:** List of COVID-19 clinical trials using immunomodulatory therapies.

**Category**	**Therapeutic(s)**	**Trial identifier**	**Phase**	**Location (centers)**	**Patients (*n*)**	**Study eligibility**	**Mechanism of action(s)**
**NK cell-based products**							
Adoptive NK cells	NK cells	NCT04280224	I	Henan, China	30	Pneumatic COVID19+	Adoptive NK cell therapy
	CYNK-001	NCT04365101	I/II	New Jersey, USA	86	Mild COVID19+	From human placental CD34+ cells and culture-expanded
	NK cells isolated from healthy donor PBMCs	NCT04344548	I/II	Bagota, Colombia	10	NEWS of >4	Isolated NK cells *ex vivo* stimulated with IL-2 and IL-15
CAR-NK Cells	NKG2D-ACE2 CAR-NK cell therapy from umbilical cord blood	NCT04324996	I/II	Chongqing, China	90	Within <14 dpi	IL-15 prolongs NK cell lifespan; GM-CSF neutralizing scFV reduces recruitment of inflammatory cells; NKG2D-ACE2 CAR-NK cells can target virally infected cells; ACE2 CAR-NK can act as decoy cell
**NK cell immunostimulants**							
**Direct NK cell activation**							
IFN-α Therapy	Recombinant human IFN-α ± Thymosin alpha 1	NCT04320238	III	Hubei, China	2,944	Uninfected HCW	IFN-alpha boosts immune system; thymosin alpha 1 activates TLRs in myeloid and pDCs leading to NK cell activation
	Recombinant human IFN-α2β	NCT04293887	I	Hubei, China	328	Within <7 dpi	IFN-alpha activates interferon pathway
	Abidol Hydrochloride ± IFN atomization (Peg-IFN-α-2b)	NCT04254874	IV	Hubei, China	100	Pneumatic COVID19+	
	Rintatolimod ± Recombinant IFN-α2β	NCT04379518	I/II	New York, USA	80	Mild to moderate COVID19+	
IFN-β Therapy	Lopinavir/ritonavir ± IFN-β-1a	NCT04315948	III	France (3)	3,100	Moderate/Severe	IFN-beta activates interferon pathway
	Base therapy ± IFN-β-1a	NCT04350671	IV	Tehran, Iran	40	Within <10 dpi	Base therapy = Hydropinchloroquine + Lopinavir/Ritonavir
	Base therapy ± IFN-β-1b	NCT04276688	II	Hong Kong, HK	127	NEWS of ≥1	Base therapy = Lopinavir/ritonavir/Ribavirin
	IFN-β-1a vs. IFN-β-1b (+Base therapy)	NCT04343768	IV	Tehran, Iran	60	Moderate/Severe	Base therapy = Hydropinchloroquine + Lopinavir/Ritonavir
**Indirect NK cell activation**							
IFN-λ Therapy	Peg-IFN-Lambda-1a	NCT04331899	II	California, USA	120	Early COVID19+	IFN-lambda to boost NK cells indirectly through Monocytes, Macrophages, and pDCs IL-12 secretion
	Peg-IFN-Lambda-1a	NCT04343976	II	Boston, USA	40	Early COVID19+	
	Peg-IFN-Lambda-1a	NCT04354259	II	Toronto, Canada	140	Ambulatory and hospitalized	
	Peg-IFN-Lambda-1a	NCT04388709	II	New York, USA	66	Non-ICU COVID19+	
	Peg-IFN-Lambda-1a	NCT04344600	II	Maryland, USA	164	Asymptomatic COVID19+	
**Immunoregulatory therapies**							
Immune checkpoint blockade	Anti-PD-1 (vs. Thymosin)	NCT04268537	II	Nanjing, China	120	Respiratory failure within <48h of ICU	Prevent T-cell regulation by blocking PD-1; in COVID19+ advanced or metastatic cancer patients;
	Anti-PD-1 (pembrolizumab) + tocilizumab	NCT04335305	II	Nanjing, China	24	Pneumatic COVID19+	Tocilizumab = anti-IL-6R
	Anti-PD-1 (nivolumab) vs. GNS651	NCT04333914	II	France (4)	273	Early COVID19+	GNS651 = Chloroquine analog
**Non-specific innate immune stimulation**							
Heterologous vaccines	BCG	NCT04328441	III	Netherlands (9)	1,500	Uninfected HCW	Trains and primes innate immunity against subsequent non-specific pathogen infection
	BCG (Danish strain)	NCT04327206	III	Australia (8)	4,170	Uninfected HCW	
	BCG (Danish strain)	NCT04350931	III	Egypt	900	Uninfected HCW	
	BCG (Danish strain)	NCT04379336	III	South Africa	500	HCW	
	BCG (Tice strain)	NCT04348370	IV	USA (5)	700	Uninfected HCW	
	BCG	NCT04369794	IV	São Paulo, Brazil	1,000	Early COVID19+	
TLR agonists	PUL-042 (CpG ODN)	NCT04313023	II	Not listed	200	Unifected/asymptomatic	Activates TLR2/6/9 leading to innate immune stimulation
	PUL-042 (CpG ODN)	NCT04312997	II	Texas, USA	100	Within <7 dpi	
**Natural health products and alternative medicines**							
Vitamins	Vitamin C	NCT04323514	N/A	Palermo, Italy	500	Pneumatic COVID19+	General immune boosting properties of vitamin C and natural health products
	Vitamin C	NCT04264533	II	Hubei, China	140	ICU COVID19+	
	Vitamin C	NCT03680274	III	Quebec, Canada	800	ICU COVID19+	
	Vitamin C	NCT04344184	II	Virginia, USA	200	ICU COVID19+	
	Vitamin C + Zinc	NCT04342728	N/A	Ohio, USA	520	Outpatient COVID19+	
	Hydroxychloroquine; Azithromycin; Vitamin C, D; and Zinc	NCT04334512	II	California, USA	600	Low risk COVID-19+	
	Hydroxychloroquine; vitamin C, D; and Zinc	NCT04335084	II	California, USA	600	Uninfected HCW	
	Vitamin D	NCT04334005	N/A	Spain (2)	200	Non-severe COVID19+	Vitamin D is immunomodulatory and prevents nutritional deficiencies.
	Zinc + Vitamin D (cholecalciferol)	NCT04351490	N/A	Lille, France	3,140	Asymptomatic COVID19+	To treat zinc and vitamin D deficiency and reduce inflammation and ARDS
	High dose Vitamin D (4X)	NCT04344041	III	France (9)	260	Severe COVID19+	

*NEWS, National Early Warning Score; HCW, Healthcare Workers; Peg, pegylated; dpi, days post-infection; As of June 1, 2020*.

**Table 2:** List of COVID-19 clinical trials investigating immunotherapies for mitigating immunopathology.

**Category**	**Therapeutic(s)**	**Trial identifier**	**Phase**	**Location (centers)**	**Patients (*n*)**	**Study eligibility**	**Mechanism of action(s)**
**Anti-cytokine therapy**							
Anti IL-6	Tocilizumab	NCT04315480	II	Italy	38	Pneumatic COVID19+	Anti-IL6R mAB to prevent virus-related cytokine storm and reduce symptoms of severe COVID-19
	Tocilizumab	NCT04317092	II	Italy (27)	400	Pneumatic COVID19+	
	Tocilizumab	NCT04320615	III	Not listed	330	Pneumatic COVID19+	
	Tocilizumab	NCT04332913	N/A	Italy	30	Pneumatic COVID19+	
	Tocilizumab	NCT04335071	II	Switzerland (3)	100	Pneumatic COVID19+	
	Tocilizumab	NCT04322773	II	Denmark (2)	200	Pneumatic COVID19+	I.V. vs. S.C. routes of administration
	Tocilizumab	NCT04331795	II	Illinois, USA	50	COVID19+, not on ventilator	Low (80mg) vs. standard dose (200mg) in non-critically ill patients
	Tocilizumab	NCT04346355	II	Italy (24)	398	Pneumatic COVID19+, non-ICU	Early administration of tocilizumab on reduced ventilation time
	Tocilizumab	NCT04331808	II	Paris, France	240	Group 1: non-ICU; Group 2: ICU	
	Tocilizumab (vs. CRRT)	NCT04306705	N/A	Hubei, China	120	Pneumatic COVID19+	CRRT = continuous renal replacement therapy
	Tocilizumab (vs. Anakinra)	NCT04339712	II	Greece (17)	20	Pneumatic COVID19+, non-ICU	Anakinra - IL1r antagonist
	Tocilizumab (vs. GNS651)	NCT04333914	II	France (4)	273	Pneumatic COVID19+	Efficacy in COVID19+ advanced or metastatic cancer patients; GNS651 = chloroquine analog
	Sarilumab	NCT04315298	II/III	USA (57)	400	Pneumatic COVID19+	Low vs. high dose of sarilumab
	Sarilumab	NCT04324073	II/III	France (4)	239	Group 1: non-ICU; Group 2: ICU	
	Sarilumab	NCT04327388	II/III	Canada+France (8)	300	Pneumatic COVID19+	
	Hydroxychloroquine + Axithromycin ± Tocilizumab	NCT04332094	II	Barcelona, Spain	276	Early COVID19+, not on ventilator	
	Tocilizumab + Favipiravir	NCT04310228	N/A	China (11)	150	COVID19+	
	Tocilizumab + Anakinra + Siltuximab	NCT04330638	III	Belgium (9)	342	Pneumatic COVID19+	Anakinra - IL1r antagonist and Siltuximab - IL6r antagonist
Anti-IL-8	Anti-IL-8 (BMS-986253)	NCT04347226	II	New York, USA	138	Pneumatic COVID19+	Prevent recruitment of inflammatory cells
Anti IL-1R/Anti IFNγ	Anakinra vs. Emapalumab	NCT04324021	II/III	Italy (4)	54	Pneumatic COVID19+	Anakinra (IL1r antagonist); Emapalumab (anti-IFNγ)
	Anakinra ± Ruxolitinib	NCT04366232	II	France (3)	54	Severe COVID19+	
Anti-GM-CSF	Mavrilimumab	NCT04337216	II	Virginia, USA	10	Pneumatic COVID19+	GM-CSF is one of the main mediators of CRS in severe COVID19 patients
Jak Inhibitor	Baricitinib	NCT04399798	II	Pavia, Italy	13	Pneumatic COVID19+	Inhibits JAK1-/JAK2-mediated cytokine release and TNF-alpha
	Ritonavir ± Baricitinib	NCT04320277	III	Tuscany, Italy	60	Moderate/pneumonia COVID19+	
Extracorporeal adsorption	CytoSorb absorber	NCT04324528	N/A	Freiburg, Germany	30	Pneumatic COVID19+	“Absorbs” IL-6 in effort to reduce inflammation in ARDS patients
**Anti-inflammatories**							
Corticosteroid	Ciclesonide ± Hydroxychloroquine	NCT04330586	II	Seoul, Korea	141	Early COVID19+ (within 7 days)	Ciclesonide is an anti-inflammatory corticosteroid
	Prednisone	NCT04344288	II	Bron, France	304	Pneumatic COVID19+	Prednisone is an anti-inflammatory corticosteroid
	Hydrocortisone	NCT04348305	III	Denmark	1000	Pneumatic COVID19+	Low-dose hydrocortisone for 7 days
	Dexamethasone	NCT04344730	N/A	Paris, France	550	COVID19+ ICU within 48hrs	Dexamethasone is an anti-inflammatory corticosteroid
	Dexamethasone	NCT04325061	IV	Spain (24)	200	Severe COVID19+ on ventilator	
	Dexamethasone	NCT04327401	III	Brazil (21)	290	ARDS patients	
	Methylprednisolone	NCT04343729	II	Brazil	420	Pneumatic COVID19+	Methylprednisolone is an anti-inflammatory corticosteroid
	Methylprednisolone (vs. Tocilizumab)	NCT04345445	III	Kuala Lumpur, Malaysia	310	COVID19+ with high risk of CRS	
	Methylprednisolone (vs. Siltuximab)	NCT04329650	II	Barcelona, Spain	200	Pneumatic COVID19+	
NSAID	Naproxen	NCT04325633	III	Paris, France	584	Pneumatic COVID19+	COX-2 inhibitor
	Ibuprofen	NCT04334629	IV	Not listed	230	NEWS2 > 5 overall, Pneumatic	

*NEWS, National Early Warning Score; HCW, Healthcare Workers; As of June 1, 2020*.

### NK Cell-Based Products

In the absence of a clinically approved vaccine against SARS-CoV-2, scientists have begun developing therapeutics to halt the spread of COVID-19 by alternative strategies. Studies have reported that patients infected with SARS-CoV-2 have lower levels of circulating NK cells and these express a greater level of inhibitory receptors (e.g., NKG2A) while producing less IFN-γ (127, 129, 130). These findings provide a rationale for pursuing NK cell-based therapies as a tool to fight COVID-19. Although NK cell-based therapies have mostly been developed for use against cancer, similar concepts and mechanisms could provide guidance in the fight against viruses.

Therapeutic NK cell products can be thought of as “living drugs” as they generally use either primary NK cells isolated from peripheral blood mononuclear cells (PBMCs) or are generated from stem cell precursors or genetically engineered immortalized human NK cell lines (158). Primary NK cell products are often pre-treated and expanded *in vitro* with cytokines or via co-culture with target cells before being infused into patients. Patients can also receive immune stimulants [e.g. recombinant IL-2 (159) or IL-15 (160)] with the goal of improving the *in vivo* activity and persistence of the NK cell products (161) as is being tested in this COVID-19 trial (NCT04344548). The first cell-based investigational drug to be approved by the FDA for clinical testing in COVID-19 patients is an allogeneic, off-the-shelf, cryopreserved NK cell therapy made by Celularity (CYNK-001), originally developed for cancer immunotherapy (162). The trial (NCT04365101) is split into two Phases. Phase I will assess the frequency and severity of adverse events in mild, non-ICU COVID-19 patients (*n* = 14) following infusion of NK cells derived from placental CD34^+^ cells. The subsequent Phase II trial will recruit up to 72 patients and include a standard of care comparator at a 1:1 allocation.

Genetically modified NK cells are also being investigated for efficacy against COVID-19. Chimeric antigen receptor NK cells (CAR-NK cells) are engineered to express virtually any receptor(s) of interest and were originally designed to enhance the ability of NK cells to eliminate cancer cells via receptors targeting EGFR (163) or CD19 (164), which are present on many cancer types and B cell hematological malignancies, respectively (164). Although the efficacy of CAR-NK cells to control viral infections has yet to be rigorously tested in large scale clinical trials, the promising safety profile of CAR-NK cells in cancer patients, who are often immunocompromised, suggests that CAR-NK therapy can be well-tolerated in early phase/mild COVID-19 patients. Notably, CAR-NK cells are considered “safe” largely because they are less likely to lead to cytokine release syndrome (CRS), a severe adverse event of CAR-T cell therapy (165). But as these are unchartered waters, it is critical that CAR-NK cells are used cautiously and not given to late/severe COVID-19 patients.

A Phase I/II study in early stage COVID-19 patients (within 14 days of illness) employing CAR-NK cell therapy is currently being tested using off-the-shelf NK cells derived from human umbilical cord blood expressing NKG2D and ACE2 CARs (NCT04324996). This complex five-arm study will compare the efficacy of different CAR-NK constructs: (i) NK cells, (ii) NK cells secreting IL-15, (iii) NKG2D CAR-NK cells, (iv) ACE2 CAR-NK cells, and (v) NKG2D-ACE2 CAR-NK cells. NKG2D CAR-NK cells have shown promising preclinical results in cancer studies (166), and although not proven for SARS-CoV-2, the rationale for expressing NKG2D derives from work showing that NKG2D-ligands (NKG2DL) are upregulated on virally infected cells (167). Similarly, the investigators hypothesize that expressing ACE2 on NK cells will facilitate the elimination of SARS-CoV-2 virions and infected cells by binding the viral spike proteins–but it is unknown whether or not CAR-NK cells can eliminate virions or if infected cells display sufficient levels of spike protein to be recognized by ACE2-NK cells upon viral infection. The investigators also suggest that expressing ACE2 on NK cells may also have a secondary benefit as a decoy cell that will be infected by the virus thereby indirectly protecting lung epithelial cells. As described previously, it is unclear whether this strategy will work to stop viral spread to healthy epithelial cells or if it will serve to perpetuate viral spread if the virus can replicate in NK cells. In arms ii-v of this trial, the CAR-NK cells have been engineered to secrete IL-15 based on studies showing improved *in vivo* persistence of CAR-NK cells in cancer patients (168). However, the addition of the proinflammatory cytokine IL-15 to this treatment strategy should be monitored closely for life-threatening toxicities, as elevated IL-15 has been previously reported to accompany chronic pulmonary inflammatory diseases (169) and MERS-CoV infection (170) even if no correlation has been reported for SARS-CoV-2. Interestingly, a study compared IL-15 levels from lung tissue homogenates following SARS-CoV infection in aged vs. juvenile monkeys and showed that IL-15 concentrations were only elevated in juvenile monkeys 10 days post-infection (171). This study would suggest that IL-15 therapy may be tolerated and effective in older COVID-19 patients that may not be able to produce IL-15, however this has not been confirmed. Lastly, all the CAR-NK cells in this trial secrete GM-CSF neutralizing scFv antibodies, since this cytokine has a known role in CRS in cancer patients treated with CAR-T cells (172), and has been shown to be correlated with COVID-19 disease severity in association with pathogenic CD4^+^ Th1 cells (173).

Although NK cell based therapies are versatile, have shown safety and efficacy in cancer patients, and can be utilized in immunocompromised individuals, their potential has yet to be fully realized as an antiviral therapy. Furthermore, the logistics of manufacturing NK cell products (cost and time) may pose limitations and barriers to access. For this reason, therapies focused on stimulating a patient's own NK cells offer many advantages over adoptive transfer of NK cells.

### Interferon Therapy and NK Cells

The importance of the interferon pathway is underscored by the fact that many viruses actively interfere with host interferon responses, for which coronaviruses are a prime example. As described above, CoVs utilize numerous tactics to avoid elimination by disrupting the host type I IFN response (174). Therefore, since the majority of CoVs fail to induce any detectable type I IFN response, eliciting a type I IFN response is a very attractive therapeutic strategy (118, 175).

Given the robust immunomodulatory nature of type I IFNs, uninfected or early symptomatic patients would benefit the most from this therapy to prevent exacerbating immunopathology at later stages of disease. Numerous clinical trials have been initiated investigating type I IFNs (Table 1). A large study (NCT04320238) of ~3,000 medical staff allocated participants to two trial arms: (i) low-risk (non-isolated wards or laboratories) or (ii) high-risk (isolated wards in direct contact with COVID-19 patients). In addition to the IFN-α-1b nasal drops, high-risk medical staff will also receive the immune-modulating TLR activator, thymosin α1, which indirectly activates NK cells through pDCs (176, 177). Interestingly, reports in SARS-CoV-1 studies showed that IFN-β therapy had a 50-fold greater anti-viral activity in Vero cells than IFN-α treatment (178). Promising results have been published from a Phase II study (NCT04276688) (179), showing that complementing lopinavir-ritonavir and ribavirin with subcutaneous IFN-β-1b in mild-to-moderate COVID-19 patients is safe with no serious adverse events reported in the triple combination therapy group, and highly effective, with significant and clinically meaningful reductions in time to complete alleviation of symptoms, hospital length of stay, and time to negative viral load (179).

Despite our best efforts in timing type I IFN therapy to mitigate immunopathology, these treatments still increase the risk of excessive activation of proinflammatory signals, which could damage host tissues and perpetuate immunopathology (180, 181). For this reason, alternative therapeutic avenues to direct type I IFN administration are being explored.

Type III IFNs can be a valid alternative to type I IFNs, because they maintain antiviral functions yet are less toxic and less prone to mediate immunopathology (182). The type III IFN, IFN-λ, activates NK cells indirectly (compared to type I IFNs which directly act on NK cells), resulting in a less potent and slower immune response (183, 184). IFN-λ activates NK cells by stimulating macrophages to produce IL-12 which in turn induce NK cells to produce IFN-γ (185). Pegylated IFN-λ is being tested in COVID-19 positive patients with mild symptoms in the absence of respiratory distress (NCT04331899). While IFN-λ can lead to the eventual activation of NK cells, its primary utility is in preventing the tissue damaging potential of neutrophils at mucosal surfaces, such as the lungs. However, IFN-λ also has been shown to reduce the rate of tissue repair, which in the context of COVID-19 which has a long disease course, could mean greater risk of secondary infections. Since exogenous administration of any IFN therapy poses the risk of tipping the balance toward severe COVID-19 immunopathology, Broggi et al. assessed the levels of IFNs in upper and lower respiratory samples from healthy and COVID-19 patients. In this preprint, they report that while the upper airway swabs showed similar mRNA expression levels of type I and III IFN compared to healthy controls, the BALF samples of severe COVID-19 patients had significantly elevated type I and III IFN levels (186). Therefore, as with all of the therapies discussed in this review, careful consideration about safe and effective timing should guide our design of clinical trials.

### Interleukin Therapy and NK Cells

In addition to IFN cytokine therapy, interleukin cytokine therapy can enhance the effector functions of NK cells (158). The use of whole, unmodified recombinant cytokines as a monotherapy has resulted in minimal success in humans in cancer immunotherapy. The earliest cytokine therapies to gain FDA-approval were IFN-α and recombinant IL-2, approved for renal cell carcinoma and metastatic melanoma (187). Although approved, they were limited by their *in vivo* half-life, marginal anti-tumor activity, and associated toxicities. The next generation of cytokine therapies were created to address these issues by first improving their biological stability through pegylation and fusion to chaperone molecules and secondly improving their specificity by fusing cytokines with antibodies or intratumoral administration. These advances in the field have allowed for the reassessment of the therapeutic potential of specific cytokines (187).

Given the importance of IL-15 signaling and NK cell function, researchers have developed IL-15 “superagonists” which are IL-15:IL-15R heterodimers that have better *in vivo* stability and bioactivity compared to monomeric IL-15 (168). Although at the time of writing IL-15 superagonists are not being studied for their efficacy in COVID-19 patients, IL-15 superagonists, such as ALT-803, are safe in humans (188) and have been used in conjunction with many of the therapies being discussed in this review including: CAR-NK cell therapy, adoptive NK cell transfers, checkpoint inhibitors, and the BCG vaccine in cancer (189). It should be noted that although the therapeutic potential of cytokine therapy to specifically stimulate NK cells is enticing, exogenous cytokine therapy has a high risk for exacerbating CRS if given at the incorrect time.

### Checkpoint Immunotherapies and NK Cells

Some viruses are known to induce a state of functional hyporesponsiveness in T cells that is essential for the productive establishment of chronic viral infections (190). A vast body of literature has identified inhibitory checkpoint receptors, including CTLA4 and PD-1, as key regulators of this process (191). Interestingly, cancer exploits similar mechanisms to escape the immune response, which provided the rationale for the introduction of antibodies targeting checkpoint receptors for cancer immunotherapy (192). CTLA4 and PD-1/PD-L1 blockade have revolutionized cancer immunotherapy, and their success provides a strong rationale for the use of these drugs in COVID-19 patients, where emerging evidence suggests that the immune response is also subverted. A clinical trial (NCT04268537) is currently assessing the efficacy of PD-1 blocking antibodies in severe COVID-19 patients within 48 h of reported respiratory distress. PD-1 has also been shown to play a role in regulating NK cell responses, in addition to modulating T cell functions (193–197), and has been reportedly increased in COVID-19 patients (129).

Inhibitory receptors on the surface of NK cells regulate NK cell activation and can be targeted by antibody therapy. One of the most promising is certainly the inhibitory receptor NKG2A, which binds to HLA-E (74, 198, 199). NKG2A expression is increased in circulating (127) and BALF NK cells from COVID-19 patients, in contrast to NKG2C, an activating receptor closely related to NKG2A, which remains unchanged (129). However, it is unclear whether the observed increase in NKG2A^+^ NK cells is due selective proliferation of NKG2A^+^ cells or if it is the result of NKG2A negative cells migrating out of circulation to infected tissues. Circulating NK cells from patients with active hepatitis B disease had higher levels of NKG2A compared to patients without active disease, however antiviral administration was associated with a reduction in NKG2A expression. Additionally, blocking NKG2A *in vitro* with NKG2A monoclonal antibodies led to improved NK cytotoxicity (200). Given the association between NKG2A expression in patients with severe COVID-19 (127, 201), a promising avenue of investigation would be anti-NKG2A therapy, even in light of results showing that NKG2A^+^ NK cells are tuned to present a higher level of responsiveness to stimulation (202).

## Indirect NK Cell Activation Through Innate Immune Stimulation

While NK cells can be stimulated directly by cytokines such as interferons and interleukins, their activity can also be enhanced through a by-stander effect following stimulation of other innate immune cells, such as macrophages and pDCs (Table 1). This type of coordinated innate immune response may be more effective at CoV viral clearance and mitigation of severe COVID-19.

### Trained Immunity and Heterologous Vaccines

Trained immunity has been recently described as an epigenetic re-wiring occurring in myeloid cells and progenitors upon stimulation that primes for a stronger response to subsequent stimuli, even of a different nature (90, 203, 204). Whereas, the consensus is that myeloid cells are primarily responsible for trained immunity (205), it is likely that the resulting alteration in the cytokine milieu also has an effect on NK cells (204, 206). This is the case for the BCG vaccine, which has been shown to provide non-specific protection against yellow fever viral infection (90, 207, 208). The BCG vaccine is composed of a live attenuated strain of *Mycobacterium bovis* originally given to young children to protect against tuberculosis (*M*. *tuberculosis*) (209). This vaccine provides an initial boost to innate immunity, but more importantly, results in the secretion of IL-1β from monocytes/macrophages, which feeds back to further stimulate the innate response (204).

The use of a heterologous vaccine to provide enhanced protection against non-specific/new pathogens makes this a compelling strategy against COVID-19 that warrants thorough investigation in randomized controlled trials (209, 210). The BCG vaccine is undergoing clinical trials in healthcare workers in the Netherlands (NCT04328441), Australia (NCT04327206), Egypt (NCT04350931), and the USA (NCT04348370) to enhance overall innate immunity and provide heterologous protection against SARS-CoV-2. Interestingly, an association was found that linked lower COVID-19-attributable mortality rates in countries using BCG in their national immunization schedules (211). On the contrary, a study that assessed the association of childhood BCG vaccination in adults living in Israel did not show a beneficial difference in COVID-19 infection rates. The discrepancy between these two reports likely stem from the fact that the latter study only included adults who were previously vaccinated during childhood, supporting the fact that heterologous vaccination may not result in long-term protection (212). Childhood BCG immunization has a limited window of opportunity to protect younger individuals from infection (213), but it is hypothesized that reducing the number of infected children can have a meaningful impact on curbing the spread of COVID-19 to the rest of the population (206, 211). Another heterologous vaccine in the process of clinical trial development for COVID-19 studies is IMM-101 (CCTG ID# IC8). Created by Immodulon Therapeutics LTD, IMM-101 is composed of heat-killed *Mycobacterium obuense* and may have an improved safety profile over the BCG vaccine (214). IMM-101 has been studied in multiple clinical trials for its non-specific immune stimulating properties as a cancer immunotherapy in pancreatic (215) and melanoma patients (216, 217).

### Toll-like Receptor Agonist Therapy

Agonists of Toll-Like Receptors (TLRs) have been shown to broadly activate different immune populations and have had both preclinical and clinical success as adjuvants in vaccination and in the treatment of a variety of viral pathogens (218). For example, CpG oligodeoxynucleotides (CpG ODNs) are short DNA sequences that contain unmethylated CpG dinucleotides which activate TLR9 particularly on DCs and B cells (219). Bao et al. showed that their CpG ODN construct, BW001, had protective effects against SARS-CoV-1 in a mechanism that relied on NK cell activation likely through a DC intermediate (220). Amidst the ongoing SARS-CoV-2 pandemic, two clinical trials (NCT04313023, NCT04312997) have opened using the TLR2/6/9 agonist, PUL-042, in order to prevent infection.

### Immune-Boosting Natural Health Products

Ascorbic acid, more commonly known as vitamin C, has been shown to exhibit potent immunomodulatory, antioxidant, and antimicrobial effects (221). Vitamin C has been shown to restore NK cell cytotoxicity in individuals exposed to toxic chemicals through protein kinase C expression, a critical component in lymphocyte metabolism (222). Additional reports have shown that vitamin C also enhances the expression of NKp46, CD69, CD25 and IFN-γ production by NK cells (223) and can increase the expression of IRF3 in lung tissues of influenza infected, pneumonia-induced mice (224). Vitamin C also harbors potent antioxidant attributes which can scavenge reactive oxygen species (ROS) and prevent lung injury (225, 226). Although ROS production is an important component in the host defense response to viruses, they can be harmful to cells and lead to the pathogenesis of viral-induced host injury (227).

The underlying rationale to investigate the therapeutic potential of vitamin C has been based on two key observations: (i) critically ill patients have lower levels of vitamin C (228–230) and (ii) vitamin C has pleiotropic immunomodulatory, antioxidant, and antiviral effects (221). It is important to underscore that reports on the clinical outcomes of vitamin C treatment in humans are mixed and context dependent. A thorough meta-analysis on vitamin C supplementation for the common cold has been reported by Hemilä and Chalker (231). Briefly, they concluded that while the incidence of colds was not reduced, the duration and severity of colds was reduced when assessing studies of regular vitamin C intake (231). Interestingly, a separate meta-analysis on vitamin C and cardiac surgery showed a reduction in the length of ICU stay and shortened the need for mechanical ventilation (232). This is an important correlation as clinical trials are currently investigating the efficacy of vitamin C to reduce mortality and hospital burden in COVID-19 patients (Table 1). A Phase II clinical trial (NCT04264533) was initiated in Wuhan where COVID-19 patients will be given a high dose intravenous infusion of vitamin C. Lastly, whether oral dosing of vitamin C can achieve therapeutically relevant concentrations, as described in the above studies, is currently unknown, thus caution should be taken as exceeding the recommended dietary allowance of 100–200 mg/day may lead to mild toxicities including abdominal discomfort and diarrhea (231, 233).

## Mitigating Immunopathology in Severe COVID-19 Patients

The main cause of death for COVID-19 patients has been pulmonary complications and respiratory failure often as a result of an unregulated cytokine storm (234). It is unclear whether the hyperinflammation seen in severe cases of COVID-19 is the result of the viral replication within pulmonary epithelial cells or an overactive/avalanching immune response. However, studies in SARS-CoV-1 reported hyperinflammation in later stages of disease progression, despite reduced viral titers, suggesting that the damage was immune-mediated (19). The most appropriate course of therapy can only be determined by elucidating the pathophysiology of disease progression. Scientists and physicians, however, have had to respond quickly to the growing number of severe COVID-19 cases and this has resulted in therapy mainly through a combination of anti-inflammatory and anti-viral interventions (Table 2). As described above, there is a potential for NK cells to contribute to the cytokine storm and therefore the development of ALI. A possible explanation for the observed lymphopenia in COVID-19 patients is that NK cells and other lymphocytes migrate out of the circulation and into pulmonary tissues to aid in the elimination of infected epithelial cells (235). This could be the premise for the large, unintended, amount of tissue damage that worsen the respiratory distress (148). For this reason, therapeutics that dampen the immune response have been effective in mitigating immunopathology in severe COVID-19 patients. The following review papers have thoroughly discussed many of these immunotherapies already (236–241), therefore, this section will focus on immunotherapies and their potential implications on NK cells.

### Anti-cytokine Therapy

The main cytokines responsible for the life threatening respiratory distress seen in reported cases of severe COVID-19 are IL-2, IL-6, IL-7, IL-10, G-CSF, IP-10, MCP-1, MIP1A, and TNF-α (234). Many clinical trials have focused on targeting IL-6 signaling with anti-IL-6R monoclonal antibodies (e.g., tocilizumab, sarilumab, siltuximab) because of the important role IL-6 has in propagating CRS (242). Tocilizumab, in particular, is being used as the primary therapy in the majority of these trials, likely owing to its FDA approved status as a therapeutic for CRS in CAR-T cell therapy (243). A case report demonstrated the potential for tocilizumab therapy in treating severe COVID-19 illness, where a single dose on day 24 of symptoms led to progressive reduction in IL-6 levels and resolution of symptoms (244). A Phase III study (NCT04320615) led by Hoffman-La Roche is recruiting patients to study the safety and efficacy of tocilizumab therapy in a randomized, double-blind, placebo-controlled, multicenter study in over 300 patients with severe COVID-19 pneumonia (Table 2). Targeting the IL-6 axis in severe COVID-19 patients may also serve to improve NK cell functions as Cifaldi et al. showed that increased IL-6 negatively impacts NK cell function (245). They also showed that tocilizumab treatment improved NK cell function *in vitro* (245). Mazzoni et al. recently reported that serum IL-6 levels were inversely correlated (*p* = 0.01) with NK cell function in COVID-19 ICU patients. Additionally, in a small subset of COVID-19 ICU patients (*n* = 5), NK cells displayed improved markers of activation (granzyme A and perforin) after tocilizumab treatment (246).

Similar therapies have emerged in the fight against COVID-19 including an IL-1R antagonist (Anakinra; NCT04330638) (247) and Cytosorb (NCT04324528) (248). High dose anakinra therapy has shown promising safety and efficacy in a small retrospective study, as part of the COVID-19 Biobank study (NCT04318366) (247). Cytosorb therapy is used in conjunction with conventional dialysis through a whole blood cartridge-based filtration system designed to remove middle molecular weight molecules (which include inflammatory cytokines <75 kDa) through extracorporeal cytokine adsorption (248). It is reported to be effective at removing Ferritin and IL-6 in a case study of a 14-year-old with severe CRS following CAR-T cell therapy (249).

Jak1/2 inhibitors (JAKi) are also undergoing clinical trials in moderate-severe COVID-19 patients, such as baricitinib (NCT04320277). In addition to their ability to impede the production of IL-6, thus curb the excessive inflammation, they may also block clathrin mediated endocytosis–indicating a dual role for JAKi (241). However, JAKi can also lead to the transient increase in NK cells as shown in baricitinib treated Rheumatoid Arthritis patients (250), which could be detrimental for severe COVID-19 patients.

### Corticosteroids

Corticosteroids have played a key role in the treatment of auto-immune diseases over the past 70 years (251, 252). Whether endogenous or exogenous, corticosteroids decrease the number of circulating monocytes and lymphocytes and decrease synthesis of pro-inflammatory cytokines (IL-2, IL-6, TNF-α) (251). Their strong anti-inflammatory and immunosuppressive effects make them good candidates for rapidly suppressing inflammation during early auto-immune disease or viral infections. Corticosteroids have been shown to inhibit NK cells in *ex vivo* experiments (253, 254). While corticosteroids may delay clearance of infections, their major benefit lies in suppressing excessive innate immune responses, thus preventing lung damage and ARDS commonly present in severe viral infections (255–257). In fact, this was the main rationale for the widespread use of corticosteroids during MERS and SARS infections (255, 256). Specific to COVID-19, some groups have advocated for the use of low-dose corticosteroids in a specific subset of critically-ill patients with refractory ARDS, sepsis, or septic shock (Table 2) (257). There is one known ongoing randomized clinical trial examining the effect of the corticosteroid ciclesonide in adults with mild COVID-19 infections (NCT04330586). This trial is based on preclinical studies showing *in vitro* antiviral activity of ciclesonide against SARS-CoV-2.

While there may be a benefit to using corticosteroids in a subset of critically-ill patients with refractory ARDS or sepsis (257), their routine use in COVID-19 is not recommended outside of clinical trials, based on expert opinion and WHO recommendations (258–260). Corticosteroids also cause a multitude of side effects, most notably diabetes mellitus, osteoporosis, and increased risk of infections (251). Controversially, a 2019 systematic review of over 6,500 influenza patients showed that corticosteroids actually led to increased mortality, length of ICU stay, and secondary infections (261). Additionally, one retrospective observational study examined the use of corticosteroids in 31 COVID-19 patients, and reported no significant association between corticosteroids and viral clearance time, hospital length of stay, or duration of symptoms (262). These studies highlight the need to be vigilant in our attempts to fight COVID-19.

### Non-steroidal Anti-inflammatory Drugs (NSAIDs)

Non-steroidal anti-inflammatory drugs, or NSAIDs, are one of the most commonly prescribed drugs for treating fever, pain, and inflammation. NSAIDs include over-the-counter household names such as ibuprofen, naproxen, and aspirin. Given the widespread use of these medications it is appropriate that researchers have investigated the potential benefits and harms of NSAIDs in patients diagnosed with COVID-19. Thus far, the evidence for using NSAIDs in the context of CoVs are mixed and might not be generalizable to all NSAIDs as reports tended to focus on specific NSAIDs. These studies also focused on the potential for NSAIDs to act as an antiviral, with a potential added benefit of being able to treat inflammatory symptoms. One report showed that the NSAID indomethacin could directly inhibit SARS-CoV replication in Vero cell monolayers in a dose-dependent manner (263). The antiviral properties of naproxen have been described in the context of influenza virus (264, 265) and has prompted the initiation of a clinical trial investigating the efficacy of naproxen as a treatment for critically ill COVID-19 infected patients (NCT04325633).

NSAID therapy should be used with caution as they have been shown to interfere with immune responses and ability to produce antibodies, with ibuprofen having the greatest suppressive effect (266). Furthermore, ibuprofen has been reported to increase the expression of the ACE2 receptor (267) which could facilitate SARS-CoV-2 viral entry. This finding should be considered for any current (NCT04334629) and potential COVID-19 clinical trial assessing ibuprofen therapy. NSAIDs also have been shown to have a direct suppressive effect on NK cell IFN-γ and TNF-α production (268) which may be beneficial for late stage COVID-19 patients.

## Conclusions and Further Study

**Figure 2:**
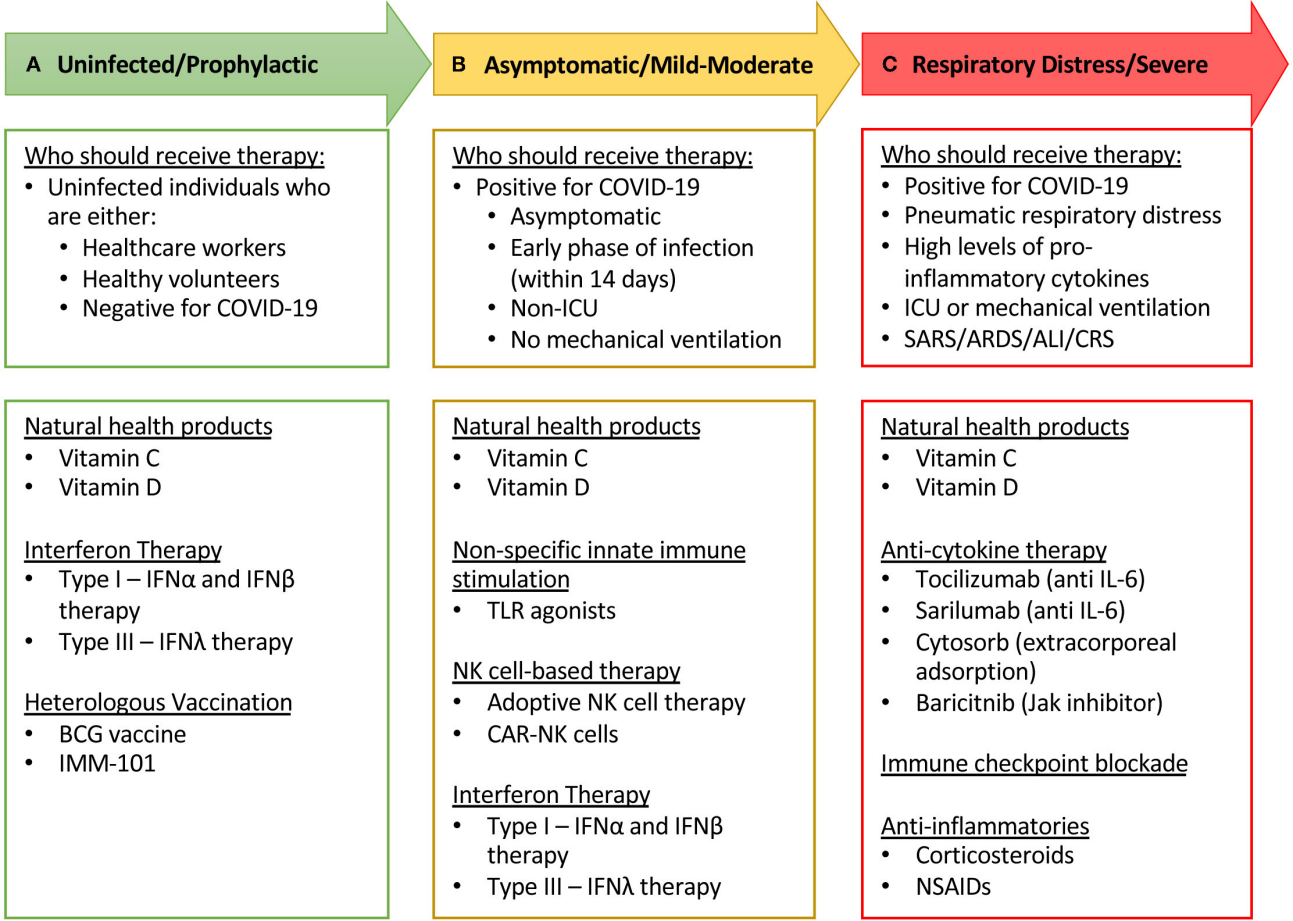
Short-list of immune modulating therapies undergoing clinical trial in COVID-19 patients and recommendations on who should receive therapy. **(A)** Healthy, uninfected individuals, who are at a high risk of becoming infected (through situational circumstances such as healthcare workers) would be most fit and suitable to receive investigational prophylactic therapies such as exogenous IFNs and heterologous vaccines. **(B)** Individuals who have tested positive for COVID-19 that are asymptomatic or have mild to moderate disease progression may benefit from receiving investigational immune stimulating therapies, including NK cell-based therapies. It is critical that investigators must be vigilant to assess the safety profile and potential immunopathologies associated with these immunotherapies. **(C)** In severe COVID-19 patients, the most appropriate therapies to investigate would be those that mitigate immunopathologies, such as anti-inflammatory and immunosuppressive therapies. Given the relatively low chance of toxicity and the wide range of beneficial immune effects, natural health products such as vitamin C and vitamin D can be suitable for investigation at all categories of COVID-19 patients.

The relevance of NK cells as antiviral first responders is highlighted in patients with NKD and immunocompromised individuals who show increased susceptibility to viral infections. While there is currently little direct evidence to support a role for NK cells in the clearance of SARS-CoV-2 there is a paucity of research in this field. However, studies in admitted COVID-19 patients with mild and severe disease reported a reduction in circulating NK cell levels and function as compared to healthy individuals. Furthermore, reduced NK cell levels and function were inversely correlated with disease severity, suggesting that NK cells may be involved in some capacity. One of the potential mechanisms by which NK cells may become hyporesponsive is via SARS-CoV-2 interference with type I IFN pathways. In investigating the pathogenesis of other CoV infections, namely SARS and MERS, studies suggest that during acute CoV infection, inflammatory monocyte-macrophages and neutrophils accumulate in the lungs and produce chemokines and cytokines that induce NK cell migration and activation. As NK cells are one of the main producers of IFN-γ, they may be involved in the IFN-γ-led cytokine storm that is responsible for the induction of inflammation-mediated ALI, ARDS, and subsequent mortality associated with COVID-19. Inarguably, more research into the role of NK cells in COVID-19 is required. Despite the knowledge gaps in COVID-19 pathophysiology, there has been a surge of clinical trials as the FDA continues to fast-track the approval of investigational therapeutics (269). Here we have outlined potential therapeutics with a focus on mediating NK cell activity, including prophylactic treatments that could boost innate immunity in addition to therapeutics that could mitigate the immunopathological consequences of COVID-19, thereby relieving the burden on our health care systems (Figure 2). Rigorous preclinical testing and thoughtfully designed clinical trials will be necessary for the development of robust therapeutics against SARS-CoV-2. Importantly, we must be aware of the potential dangers immunotherapies may have in potentiating CoV immunopathology. The fight against COVID-19 is not an easy one. As with any novel disease, we will have to rely on incomplete pictures to guide reasoning for appropriate treatment. We believe that immunotherapeutics targeting the innate immune response, and specifically NK cells, have the potential to flatten the curve and will be important instruments in our armamentarium against this pandemic and the next.

## Author Contributions

MM, LA, AM, DB, OO, GT, DMB, and JN contributed to literature review. MM, LA, AM, DB, OO, and GT contributed to writing. MM, LA, MA, and RA were responsible for editing. MM and LA were responsible for formatting. MA and RA oversaw the writing process and provided mentorship and guidance. All authors contributed to the article and approved the submitted version.

## Conflict of Interest

The authors declare that the research was conducted in the absence of any commercial or financial relationships that could be construed as a potential conflict of interest.
